# Effects of Natalizumab Treatment on Foxp3+ T Regulatory Cells

**DOI:** 10.1371/journal.pone.0003319

**Published:** 2008-10-06

**Authors:** Max-Philipp Stenner, Anne Waschbisch, Dorothea Buck, Sebastian Doerck, Hermann Einsele, Klaus V. Toyka, Heinz Wiendl

**Affiliations:** 1 Department of Neurology, Julius-Maximilians University, Wuerzburg, Germany; 2 Department of Internal Medicine II, Julius-Maximilians University, Wuerzburg, Germany; New York University School of Medicine, United States of America

## Abstract

**Background:**

Natalizumab, a monoclonal humanized antibody targeting the alpha-4 chain of very late activation antigen 4 (VLA-4) exerts impressive therapeutic effects in patients with relapsing-remitting multiple sclerosis. Our objective was to study impacts of Natalizumab therapy on Foxp3+ T regulatory cells (Tregs) in multiple sclerosis (MS) patients.

**Methodology:**

A combined approach of *in vitro* and *ex vivo* experiments using T cells isolated from the peripheral blood of healthy donors and Natalizumab treated MS patients was chosen. We determined binding of Natalizumab and its effects on the frequency, transmigratory behaviour and suppressive function of Tregs.

**Principal Findings:**

Binding of Natalizumab and expression of CD49d (alpha-4 chain of VLA-4) differed between non-regulatory and regulatory cells. Albeit Foxp3+ Tregs had lower levels of CD49d, Natalizumab blocked the transmigration of Foxp3+ Tregs similar to non-regulatory T cells. The frequency of peripheral blood Tregs was unaffected by Natalizumab treatment. Natalizumab does not alter the suppressive capacity of CD4+CD25^high^CD127^low^Foxp3+ Tregs under *in vitro* conditions. Furthermore, the impaired function of Tregs in MS patients is not restored by Natalizumab treatment.

**Conclusions:**

We provide a first detailed analysis of Natalizumab effects on the regulatory T cell population. Our prospective study shows that Foxp3+ Tregs express lower levels of VLA-4 and bind less Natalizumab. We further the understanding of the mechanisms of action of Natalizumab by demonstrating that unlike other immunomodulatory drugs the beneficial therapeutic effects of the monoclonal antibody are largely independent of alterations in Treg frequency or function.

## Introduction

Natalizumab (Tysabri®), a monoclonal humanized antibody (mAb) targeting the alpha-4 chain of α4β1 (very late activation antigen 4; VLA-4) and α4β7 integrins on the surface of leukocytes, exerts impressive therapeutic effects in patients with relapsing-remitting multiple sclerosis [1]–[3]. However, clinical efficacy is opposed to some uncertainty about the risk-benefit profile associated to treatment [4]–[6], especially given the fact that 3 cases of progressive multifocal leukoencephalopathy (PML) have been reported in association to Natalizumab treatment in the setting of clinical trials [7], [8].

It is believed that this mAB inhibits the interaction between VLA-4 on the leukocyte surface and its respective ligand on endothelial cells (VCAM-1), thereby preventing the extravasation of leukocytes into the central nervous system (CNS) [4], [9]. Besides its effects on leukocyte transmigration, Natalizumab may elicit direct effects on T cell function. The therapeutic target of Natalizumab belongs to the large group of integrins, which are considered bi-directional signalling molecules [10]. VLA-4 forms an essential part of the immunological synapse and ligation of VLA-4 was demonstrated to provide costimulatory signals to T cells [11]–[13]. However, despite strong clinical evidence for the use of Natalizumab in patients, knowledge on biological effects on different immune cell populations in MS patients *in vivo* is still marginal [13]–[15].

Recent work supports the hypothesis that Natalizumab does not uniformly block lymphocyte extravasation but might act via selective modulation of its cellular targets. VLA-4 surface levels were demonstrated to differ considerably between specific immune cell subsets and analysis of cerebrospinal fluid in Natalizumab treated patients supports the idea that Natalizumab preferentially blocks extravasation of CD4+ over CD8+ T cells [13], [14]. These findings are of particular interest since a differential effect of Natalizumab on specific immune cell subsets might have major implications for the immunesurveillance of the CNS and could provide clues for the pathogenesis of opportunistic CNS infections in Natalizumab treated patients.

Thymus derived (natural) Foxp3+ Tregs are an emerging immune cell population crucial for the maintenance of immune-tolerance and pivotal for the control of autoimmune and infectious disorders [16], [17]. If and how Natalizumab affects this regulatory T cell population is currently unknown. We here prospectively studied how Natalizumab binds to Tregs and delineated the effect on frequency, migratory behaviour and suppressive function of Foxp3+ T regulatory cells and their non-regulatory counterparts.

## Results

### Natalizumab binding to different T cell subsets

**Figure 1:**
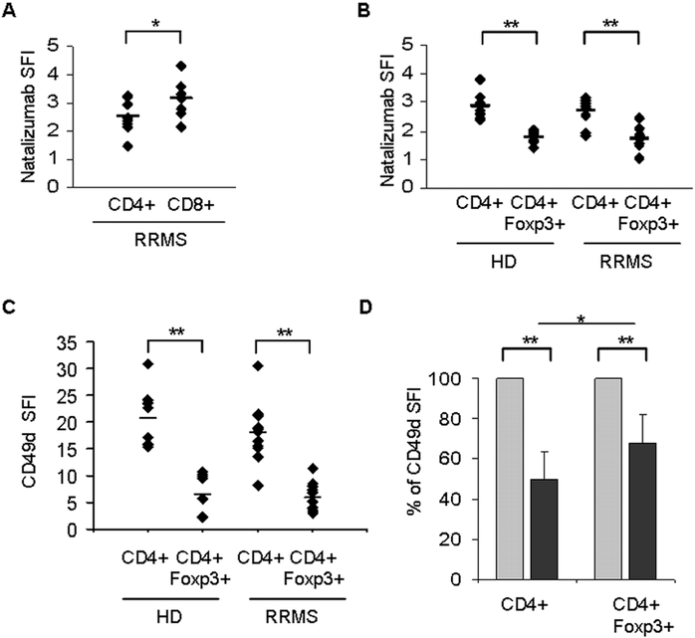
Differential binding of Natalizumab to T cell subsets. (A) Binding of FITC-conjugated Natalizumab (20 µg/ml) to CD4+ and CD8+ T cells derived from patients with a relapsing-remitting disease course (RRMS; n = 9) and (B) to regulatory, Foxp3+ and non-regulatory, Foxp3− CD4+ T cells from healthy donors (HD; n = 9) and RRMS patients (n = 10) as analyzed by flow cytometry. (C) Differences in Natalizumab binding closely reflect surface expression patterns of the α4 chain (CD49d) of VLA-4 as analyzed by flow cytometry. (D) CD49d surface levels were analyzed by flow cytometry before (light grey) and 1 month after initiation of therapy with Natalizumab (dark grey). Postinfusion levels were normalized to the expression of CD49d before treatment. The relative loss of functional VLA-4 is lower in CD4+Foxp3+ T regulatory cells than in conventional CD4+ T cells (n = 15). SFI = specific fluorescent index, * p<0,05; **p<0,005, student's t-test.

We first assessed how Natalizumab binds to Foxp3+ Tregs versus Foxp3 negative CD4+ T cells. After evaluating the saturation of Natalizumab binding sites using FITC-conjugated Natalizumab, a saturating dose of Natalizumab (20 µg/ml) was used to assess a differential binding to T cell subsets. The concentration of 20 µg/ml lies within the range of serum trough levels of treated patients [9]. A comparison of the specific fluorescent indices (SFI) of CD4+ and CD8+ T cells in untreated MS patients revealed significantly higher binding of Natalizumab to CD8+ T cells (Figure 1a). In contrast, binding of Natalizumab to the CD4+Foxp3+ subset of regulatory T cells was significantly lower compared to their CD4+Foxp3 negative counterparts, both in healthy donors and MS patients (Figure 1b). The disease course (acute relapse versus stable; n = 5 each) had no effect on Natalizumab binding to T cell subsets derived from the peripheral blood of MS patients (data not shown).

As Natalizumab binding to the alpha-4 chain of VLA-4 and α4β7 is independent of a conformational active state, the differential binding of Natalizumab to Tregs and their Foxp3 negative counterparts should be closely mimicked by the expression levels of CD49d on the cell surface. Thus, we analyzed the surface expression of alpha-4 integrin (CD49d) by the use of a distinct antibody recognizing the B epitope of CD49d (9F10). In line with our findings on Natalizumab binding, CD49d levels on Foxp3+ CD4+ T cells were much lower compared to the non-regulatory CD4+ population (Figure 1c). There were no differences in CD49d expression levels between Foxp3+ Tregs derived from MS patients as compared to healthy donors (Figure 1c).

Previously published data suggests that the differential impact of *in vivo* Natalizumab treatment on the function of immune cells is closely reflected by a relative decrease of CD49d immunoreactivity on the surface of leukocytes after initiation of therapy [13]. *Ex vivo* analysis of CD49d surface expression revealed a significant decrease on all immune cell populations under investigation 30 days after the first infusion of Natalizumab. The relative impact of Natalizumab infusion (percent of MFI) was significantly higher for non-regulatory CD4+ T cells as compared to Tregs: Natalizumab treatment resulted in a 51% reduction of CD49d immunoreactivity on CD4+ T cells whereas Foxp3+ Tregs were less affected resulting in a 32% reduction of CD49d on the Treg surface (n = 15, Figure 1d). Conversely, there was no difference between the relative loss of surface CD49d on CD4+ and CD8+, which is in line with previously published observations by Stüve and colleagues [14] (n = 11, data not shown). Of note, the impact of Natalizumab as measured by decrease of CD49d SFI on T cells directly correlates with the presence of neutralizing antibodies against Natalizumab: one patient in our cohort showed an initial decrease of CD49d immunoreactivity at M1, followed by a return to baseline at M6 (Supplementary Figure S1). He was shown to have persisting neutralizing antibodies in parallel.

### Effects of Natalizumab on the migration of T effector cells and Tregs

**Figure 2:**
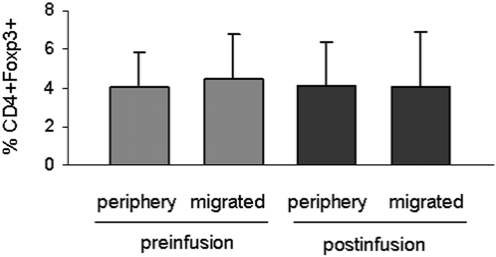
Natalizumab blocks both Tregs and non-regulatory T cells in *in vitro* transmigration assays. CD4+ T cells were analyzed for their migratory capacity in a fibronectin coated Boyden chamber assay system. The relative proportion of Foxp3+ cells before migration (periphery) and within the migrated fraction (migrated) was assessed by multicolour flow cytometry. The bar diagram demonstrates the frequency of Foxp3+ T cells in the two fractions before and after initiation of Natalizumab therapy (n = 15). No differences were observed under any condition.

It is currently unknown how the migratory behaviour differs between Foxp3+ Tregs and Foxp3− CD4+ T cells in MS patients and how blockade of VLA-4 influences migration of Tregs. According to Niino et al., the relative loss of CD49d immunoreactivity under *in vivo* therapy directly correlates with the migratory behaviour of immune cell subsets as demonstrated for monocytes, B and T lymphocytes [13]. Since expression of CD49d was significantly lower in Foxp3+ Tregs than in Foxp3− T cells and the relative decrease of CD49d immunoreactivity in the presence of Natalizumab was significantly lower on Foxp3+ Tregs compared to conventional CD4+ (Figure 1), we were curious if this finding might be associated with an aberrant migratory behaviour. To assess transmigration of CD4+ T cells we used fibronectin coated Boyden chambers, a well characterized experimental set-up to assess transmigration mediated by interaction of VLA-4 on the T cell surface with its alternative binding partner, the CS-1 fragment of fibronectin [13], [20]. In line with previous findings by Niino et al. Natalizumab blocked T cell transmigration in this experimental setting (data not shown). To find out whether Natalizumab might favour the migration of Tregs, we assessed the percentage of Foxp3+ T cells at baseline and within the migrated fraction. A selective blockade of non-regulatory CD4+ compared to Tregs should have resulted in an enrichment of Foxp3+ T cells within the migrated fraction. However, the percentage of Foxp3+ T cells within the migrated fraction remained the same irrespective of Natalizumab treatment (n = 15, Figure 2).

### Foxp3+CD4+ T cell frequency in patients undergoing Natalizumab therapy

**Figure 3:**
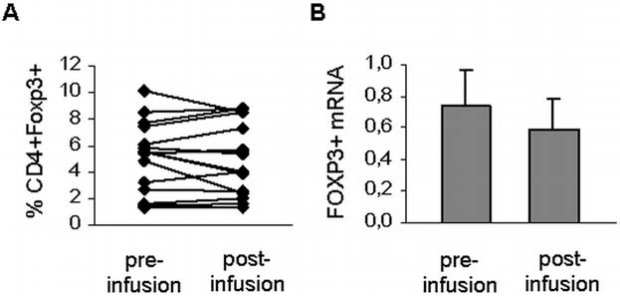
Frequency and mRNA of CD4+Foxp3+ T regulatory cells are not altered by Natalizumab therapy. (A) The frequency of CD4+Foxp3+ Tregs was determined by flow cytometry before and 30 days after the first infusion. (B) Foxp3 mRNA expression levels as analyzed by real-time PCR before and 3 months after initiation of therapy (n = 5).

VLA-4 blockade is associated with an increase of the absolute lymphocyte numbers in the peripheral blood of MS patients [2], [3], however, flow cytometrical analysis of Foxp3+ T regulatory cells in patients treated with Natalizumab demonstrated that Treg frequency was not significantly affected by therapy 30 days after Natalizumab application (n = 15, Figure 3a). In accordance with our protein data, mRNA analysis of FOXP3 expression levels on PBMC before and 3 months after the first infusion yielded comparable results (n = 5, Figure 3b).

### Natalizumab does not restore the impaired suppressive function of Tregs in MS

Besides blocking the interaction between VLA-4 and its ligand on endothelial cells, direct effects of Natalizumab on T cell function have been proposed to contribute to the beneficial effects of Natalizumab in MS. VLA-4 blockade has been reported to modulate the activation state of CD4+ T cells [13], [25]. Foxp3+ Tregs have been described to be dysfunctional in the MS patient population [23], [26], [27]. We therefore questioned whether Natalizumab may influence the suppressive function of Foxp3+ T regulatory cells. To allow a direct comparison of the suppressive capacity of MS patient derived Tregs before and after initiation of Natalizumab therapy we had to rely on frozen PBMC samples to conduct functional suppression assays. Flow cytometry of human lymphocytes thawed after cryopreservation in liquid nitrogen reveals a strong reduction in CD25 immunoreactivity as compared to freshly isolated lymphocytes ([18] and observations in our own laboratory). Sequential flow cytometrical analysis demonstrated that a 3 day culture period was necessary for the CD25 surface expression to fully recover, thus allowing the distinction of regulatory and non-regulatory T cells. Several methods of cell purification were evaluated to guarantee a high purity of Tregs. However, neither magnetic cell purification using a commercially available Treg purification kit (Miltenyi Biotec, Bergisch-Gladbach, Germany), nor fluorescence activated cell sorting for the CD4+CD25^high^ population yielded the desired purity as demonstrated by intracellular staining for Foxp3 on purified T cell subsets (Supplementary Figure S2a). However, the additional staining for the recently described marker CD127 (IL-7 receptor) distinguishing Tregs from non-regulatory cells [28], [29] and gating on the CD4+CD25^high^CD127^low^ population helped to consistently isolate highly pure (>95%) Foxp3+ Tregs by fluorescence activated cell sorting (Supplementary Figure S2b). Using this protocol, purity of the CD4+CD25^high^CD127^low^ T regulatory cells as assessed by Foxp3 expression was comparable between cryopreserved PBMC and freshly isolated cells. Parallel suppression assays using either freshly or previously cryopreserved T regulatory cells derived from the same healthy donor demonstrated that the suppressive function was not affected.

**Figure 4:**
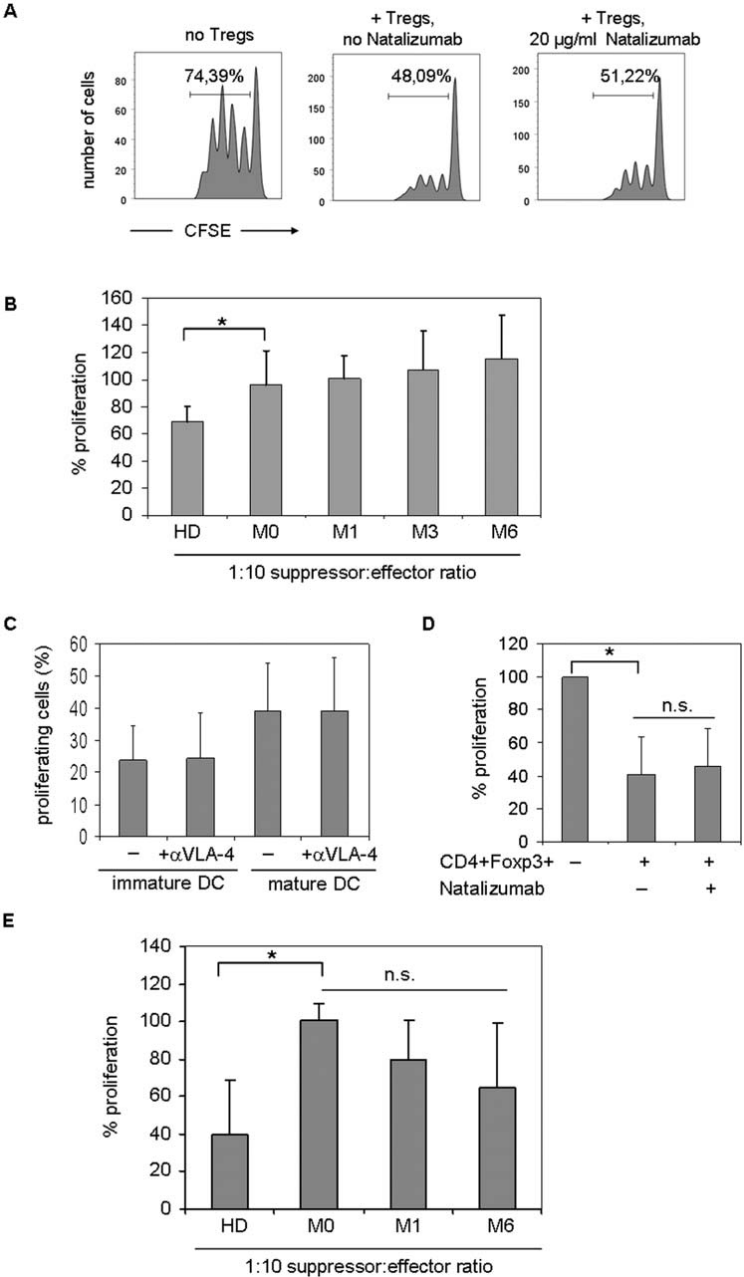
Natalizumab does not alter the suppressive capacity of CD4+CD25^high^CD127^low^ Tregs and does not restore impaired Treg function in MS. (A) CFSE-based suppression assays using fluorescence activated cell sorted T cells derived from the peripheral blood of healthy donors were conducted in the presence or absence of Natalizumab (20 µg/ml). The proliferation of responder T cells (CD4+CD25−) was suppressed in the presence of Tregs as analyzed by flow cytometry, however, the suppressive capacity of Tregs remained unaffected by the mAb. One out of 5 representative experiments is shown, the percentage of proliferating cells is indicated in the diagram. (B) Suppressive capacity of serially analyzed T cells derived from healthy donors (n = 5) and Natalizumab treated patients (n = 5 for each time point except month 6: n = 3) before (M0), 1 month (M1), 3 months (M3) and 6 months after initiation of therapy. Bars show the proliferation of responder cells (CD4+CD25−) in the presence of Tregs (1∶10 Treg to T effector ratio) after normalization to the proliferation of responder cells in the presence of an equal number of non-regulatory T cells. * p<0,05; Significant differences in suppression are observed comparing HD and RRMS Tregs at any time point during the treatment period, as exemplarily depicted for M0. (C) Percentage of proliferating T effector cells derived from healthy donors (n = 5) that were challenged by immature or mature dendritic cells (DC : T cell ratio 1∶5) in the presence or absence of Natalizumab (20 µg/ml). (D) In vitro addition of Natalizumab (20 µg/ml) does not affect Treg mediated suppression of T cell proliferation in response to immature allogeneic DC as analyzed in CFSE suppression assays. Data were normalized to the proliferation of T cells in response to DC in the presence of an equal number of non-regulatory T cells. (E) Proliferation of responder cells from healthy controls (HD, n = 4), and RRMS patients before (M0, n = 4), 1 month (M1, n = 4) and six months (M6, n = 4) after initiation of Natalizumab treatment. Normalization as in (D).

In contrast to Tregs derived from the peripheral blood of healthy donors, MS patient derived Tregs showed very poor suppression of autologous T cell proliferation as a baseline (31% versus 4% mean suppression, at a 1∶10 Treg to responder ratio; n = 5 for healthy donors and MS patients respectively). Addition of Natalizumab to the αCD3/αCD28 microbead-based suppression assays showed no significant influence on the suppressor function of Foxp3+ regulatory T cells (experiments performed with Tregs derived from healthy donors; one out of 5 representative experiment is shown, Figure 4a). Various concentrations of Natalizumab (0,5 µg/ml up to 40 µg/ml) and titration of suppressor∶effector ratios in modification of the assay conditions yielded comparable results (data not shown). We tested the effect of Natalizumab treatment on the suppressive function of Tregs in *ex vivo* αCD3/αCD28 microbead-based suppression experiments. In line with our *in vitro* data, treatment with Natalizumab did not alter the dysfunctional suppressive capacity of T regulatory cells in patients with RRMS. Over 6 months of treatment, highly pure CD4+CD25^high^CD127^low^ regulatory cells remained poor suppressors (n = 5, Figure 4b). In addition to bead-based suppression assays we used allogeneic immature and mature DC to challenge T effector cells. Since VLA-4 is part of the immunological synapse and has been suggested to be involved in costimulation [11]–[13] we first investigated whether Natalizumab influences the outcome of DC-T cell encounters by modulating T cell proliferation. However, Natalizumab (20 µg/ml) did not impact the percentage of proliferating T cells in response to immature DC (23,96+/−10,57 vs. 24,58+/−14,02) or mature DC (39,324+/−14,77 vs. 39,178+/−16,67) as determined by CFSE dilution assays (n = 5, Figure 4c). APC-based suppression assays revealed that CD4^+^CD127^low^CD25^high^ Treg cells derived from healthy donors strongly suppressed the proliferation of T effector cells challenged by allogeneic DC, as exemplarily depicted for immature DC (Figure 4c). VLA-4 blockade by addition of Natalizumab (20 µg/ml) did not affect the Treg mediated suppression in the presence of immature DC (Figure 4d). Similar results were obtained when LPS matured DC were used instead of immature DC (data not shown). Further analysis confirmed that Tregs derived from MS patients remained dysfunctional in this APC-based suppression assay (Figure 4e, n = 4). *Ex vivo* experiments using T effectors and T regulatory cells derived from Natalizumab treated patients before and after initiation of therapy (month 1 and 6) did not reveal a significant effect on the suppressive capacity of CD4^+^CD127^low^CD25^high^ Treg in the presence of allogeneic immature DC, albeit a trend towards a regain of function could be noted (Figure 4e, n = 4).

## Discussion

Our study investigated the influence of Natalizumab therapy on the Foxp3+ T regulatory cell population. Regulatory T cells recently emerged as key players in orchestrating adaptive immune responses and balancing immune tolerance versus autoimmunity [16], [30]. A role in the immune surveillance of parenchymal organs including the CNS is assumed as recent studies have suggested that Foxp3+ Tregs are capable of migrating into the CNS under inflammatory conditions and may actively modulate the function of effector cells within the target organ itself. During experimental autoimmune encephalomyelitis (EAE), peripherally expanded Tregs are found to accumulate within the CNS [31], and Foxp3+ Tregs were demonstrated to be enriched in the CSF of MS patients contributing to the hypothesis that natural Tregs may be actively “recruited” to the site of inflammation in MS [23], [32].

VLA-4 has been identified as a crucial molecule in T cell trafficking across the blood-brain barrier and VLA-4 blockade by Natalizumab efficiently inhibits migration of T effector cells into the CNS [15]. It is well perceivable that differential effects of Natalizumab on the migratory capacity or functional properties of non-regulatory and Foxp3+ T regulatory cells would have implications for CNS immunosurveillance.

Natalizumab was found to preferentially bind to T effector cells, due to a lower expression of the target molecule (alpha-4 integrin) on Tregs. This could either indicate a stronger impact of a given dose of the mAB on Tregs or they could be less prone to Natalizumab effects since other, yet unidentified molecules, might be dominant in the control of Treg trafficking. It has been suggested that instead of absolute levels of alpha-4 integrin, it is rather the relative loss of functional VLA-4 under therapy which dictates differential effects of Natalizumab on immune cell subsets^13^. In line with the literature Natalizumab therapy resulted in a marked loss of alpha-4 integrin (CD49d) immunoreactivity on all T cell subsets [13], [14]. Interestingly, the relative decrease of functional alpha-4 integrin was less pronounced in Tregs in comparison to Foxp3 negative T cells suggesting that Natalizumab may preferentially block the migration of T effector cells and thus favour the extravasation of Tregs. To test this hypothesis we chose a simplified but well characterized model system to assess migration of T cells mediated by interaction of VLA-4 with its alternate binding partner, the CS-1 fragment of fibronectin. This experimental model has previously been used for analyzing alpha-4 integrin mediated transmigration of lymphocytes [20], [21] and was found to closely and highly reliably reflect the effects of VLA-4 blockade on T cell trafficking [13]. Albeit the relative loss of functional VLA-4 was significantly lower on Tregs, Natalizumab treatment did not “favour” the transmigration of Foxp3+ Tregs. Still, due to the limitations of the chosen *in vitro* model system our data need to be interpreted carefully. Certainly, the best way to test our hypothesis would have been a detailed CSF analysis in patients under treatment, thereby comparing the frequency of Tregs within the CSF before and after initiation of therapy (e.g. at 6 or 12 months). However, we refrained from attempting this for both technical as well as medical-ethical reasons. Under VLA-4 blockade CSF cell counts are markedly diminished in treated patients [15]. Since Foxp3 cells represent a minority within the CD4 T cell populations, conclusions from flow cytometrical analysis would have been based on extremely low number of Tregs cells. Furthermore, no patient in our cohort underwent lumbar puncture for medical reasons under the prospective study period.

It has been suggested that the loss (and regain) in functional VLA-4 under therapy may be a useful biomarker to monitor individual responses to Natalizumab therapy which hold true in our study [13]. The loss of alpha-4 integrin expression was well sustained at the end of the monthly dosing interval, even after receiving only 1 infusion of Natalizumab (300 mg i.v.). Although being based on a single observation, the finding that persistent neutralizing antibodies seem to abrogate the functional loss of VLA-4 under therapy strengthens the potential role of this biomarker in monitoring Natalizumab therapy and warrants further investigations.

We challenged the influence of Natalizumab on T suppressor frequency and function. Tregs play a key role in the mechanisms of autoimmunity, a loss of Treg suppressor function seems to play a critical role in MS disease pathogenesis [23], [26], [27], [33]. Contemporary immunomodulatory drugs such as interferons and glatiramer acetate have been suggested to mediate their beneficial effects – at least partially - via modulation of Treg frequency and/or suppressive function, thereby augmenting the corollary of reconstituting Treg function as a therapeutic aim in MS [34]–[37]. Integrins including alpha-4 integrin (VLA-4) are so called bi-directional signalling molecules that connect to the cytoskeleton and activate intracellular signalling pathways [10]. Ligation of VLA-4, which forms an essential part of the immunological synapse, was demonstrated to provide costimulatory signals to T cells [11]–[13] and anti-VLA-4 therapy was suggested to modulate the activation of lymphocytes in experimental animal models of MS [25]. Accordingly, direct effects of Natalizumab on the homeostasis of Tregs and their immunobiological functions appear feasible. Our data clearly show, that the frequency of Foxp3+ T cells as well as mRNA for FOXP3 within the peripheral blood of patients was unaffected by therapy, excluding that the beneficial effects of Natalizumab are largely due to quantitative changes in the regulatory T cell population. Tregs derived from MS patients were found to be poor suppressors corroborating previously published data on a dysfunction of Tregs in RRMS by several groups including ours [23], [26], [27]. Neither in vitro VLA-4 blockade nor in vivo Natalizumab treatment restored the impaired function of Tregs in suppression assays using anti-CD3/CD28 coated bead as a stimulus. Previously Natalizumab was reported to elicit costimulatory effects [13]. Therefore, before analyzing its effects on Treg mediated suppression, we had to exclude that VLA-4 blockade interferes with T effector cell proliferation which would have biased the outcome of suppression assays. In our hands a saturating dose of Natalizumab neither enhanced nor diminished the proliferation of DC stimulated T cells. According to the literature costimulatory effects of anti-VLA-4 are subject to an inverse dose-dependency: very low doses of anti-VLA-4 (0,1 µg/ml) strongly enhance PBMC proliferation, while this effect seems to be diminished when higher doses of the mAb are employed. In our study we used 20 µg/ml which is within the range of serum trough levels of Natalizumab treated patients and saturated Natalizumab binding sites on T cells according to our own observations. To ensure the *in vivo* relevance of our findings we did not employ lower doses which may explain the lack of effects of Natalizumab on T effector cell proliferation in our experiments. Accordingly, we challenged whether Natalizumab may influence the outcome of immune responses by modulating DC Treg interactions. Although we observed a weak tendency towards an improvement of Treg function under therapy, a level of significance was not reached.

One has to mention that it may not be the entire Foxp3+ population of T regulatory that is dysfunctional in MS. A lot of research effort is currently put into identifying markers that may characterize a specific subset among Foxp3+ Tregs that is impaired in MS. Borsellino and colleagues recently reported a subpopulation of Foxp3+ Tregs with potent suppressive function identified by expression of the ectonucleotidase CD39. Although the overall frequency of Foxp3+ Tregs did not differ between healthy donors and MS patients, numbers of this specific subset were greatly reduced [38]. Similarily, Haas et al. have reported reduced numbers of a subset of naive Tregs that they defined as recent thymic emigrants by the expression of CD31. Lack of this subset was compensated for by increased numbers of memory Tregs in MS patients, suggesting that this dysequilibrium in the homeostatic composition may account for the impaired suppressive function of Foxp3+ Tregs in MS [39]. Albeit merely speculative, Natalizumab effects on a subset of Foxp3+ Tregs may have accounted for the positive trend observed in our DC-based suppression assays. Further studies unravelling Treg dysfunction by characterization of impaired subsets are eagerly awaited.

Summarizing, this is the first study prospectively analyzing the effects of Natalizumab therapy on frequency and function of regulatory T cells. Analysis of the impact of Natalizumab on immune cell function is essential not only for understanding its mechanisms of actions but also to clarify its role in the pathogenesis of progressive multifocal leukencephalopathy. We further add to the understanding of the mechanisms of action of Natalizumab by demonstrating that unlike other immunomodulatory drugs the beneficial therapeutic effects of the monoclonal antibody are largely independent of alterations in Treg frequency or function.

## Materials and Methods

### Clinical Samples

The study was approved by the local ethics committee (Ethics committee Wuerzburg) and written consent was obtained from all participants (Nr. 155/06). Natalizumab treatment was initiated in relapsing-remitting (RR)MS patients because of highly active disease that was refractory to treatment with approved disease modifying agents. Peripheral blood was obtained by vein puncture at specific time points before and after initiation of Natalizumab therapy as indicated below. All patients had been on immunmodulatory or immunosuppressive therapy prior to initiation of Natalizumab therapy. Interferon beta and glatiramer acetate were stopped at least 15 days before starting the patients on Natalizumab. Immunosuppressive therapy had to be terminated at least 6 months prior and normalization of blood counts was required before initiation of Natalizumab therapy. Patients with clinically definite RRMS either with a documented stable disease course (>6 months) or acute relapse and healthy volunteers served as a control group.

### Isolation of peripheral blood mononuclear cells, purification of T cell subsets and generation of dendritic cells

PBMC were isolated by centrifugation on a Lymphoprep™ (Fresenius Kabi Norge AS, Oslo, Norway) density gradient. To allow comparative analysis in a prospective, longitudinal study design, PBMC were immediately cryopreserved and stored in liquid nitrogen. CD4+ T cells were isolated using MACS® technology (CD4+ T cell isolation kit II, Miltenyi, Bergisch-Gladbach, Germany) according to the manufacturer's protocol.

For purification of Tregs different methods had to be evaluated. Cryopreservation has been shown to reduce CD25 immunoreactivity on Tregs [18]. Since CD25 surface expression is mandatory for purification of Tregs, PBMC were allowed to recover over a 3 day culture period, until CD25 surface levels were fully restored. PBMC were subjected to magnetic cell separation using the CD4+ CD25+ regulatory T cell isolation kit (Miltenyi) or to fluorescence activated cells sorting gating on CD4+CD25^high^ or CD4+CD25^high^CD127^low^ respectively (MoFlo Sampler, Dako, Glostrup, Denmark; FACSDiva, BD Bioscience, Heidelberg, Germany). The following antibodies were used: anti-CD4 (SK3), anti-CD127 (hIL-7R-M21), anti-CD25 (M-A251) (all from BD Biosciences, Heidelberg, Germany). Monocytes were selected by adhesion to plastic flasks for 1 h. Dendritic cells were generated as previously described .[19]. In brief, monocytes were cultured in RPMI 10% FCS supplemented with GM-CSF (100 ng/ml) and IL-4 (40 ng/ml) (both from R&D Systems, Minneapolis, MN, USA). After 5 days, the cells exhibited an immature DC phenotype as assessed by flow cytometry for CD80, CD86, MHC II and DC-SIGN. Maturation was induced by 48h-incubation of the immature DCs with LPS (5 µg/ml). High levels of surface MHC class II and costimulatory molecules (CD86, CD80), as well as low levels of DC-SIGN identified mature DCs.

### Flow cytometry

Cells were washed twice in phosphate buffered saline (PBS) containing 0.1% sodium azide and 1% bovine serum albumine, followed by Fc receptor blocking with human IgG (Sigma-Aldrich, Munich, Germany). Afterwards cells were incubated for 30 minutes with specific monoclonal antibodies. The following anti-human monoclonal antibodies were used (all fluorochrome-conjugated): anti-CD4 (SK3), anti-CD8 (SK1), anti-CD49d (9F10), anti-CD127 (hIL-7R-M21), anti-CD25 (M-A251) (all from BD Biosciences, Heidelberg, Germany) and anti-CD4 (M-T466) (from Ebioscience, San Diego, USA). The respective isotype controls (IgG1, rat IgG2a) were purchased from BD Biosciences. Intracellular stainings using anti-Foxp3 (PCH101) antibody were performed using a Foxp3 staining kit (Ebiosciences, San Diego, CA, USA) according to the manufacturer's protocol. All antibodies were titrated for optimal concentrations. A FACSCalibur flow cytometer (BD, Heidelberg, Germany) was used. Data was analyzed using FlowJo software (Tree Star Inc. Ashland, OR, USA). Histograms were analyzed by calculating the specific fluorescence index (SFI) [geometric mean of the specific antibody fluorescence divided by the geometric mean of the isotype control antibody fluorescence].

### Natalizumab binding assay

Natalizumab was labelled with fluorescein isothiocyanate (FITC) using the EZ-Label™ FITC Protein Labelling Kit (Pierce, Rockford, USA) according to the manufacturer's instructions. Cells were incubated with 20 µg/ml of Natalizumab for 30 min, followed by staining for additional surface makers and analyzed by multicolour flow cytometry. Binding of Natalizumab-FITC was completely abrogated in the presence of a 10 fold excess of unlabeled Natalizumab, which was used as a negative control in each donor to calculate the specific fluorescence index (SFI).

### Analysis of FOXP3 mRNA levels by quantitative real-time PCR

RNA was isolated using the Rneasy® Mini Kit (Qiagen, Hilden, Germany). 500 ng of mRNA were transcribed using random hexamers and MuLV reverse transcriptase (all reagents supplied by Applied Biosystems, Foster City, USA). Gene expression assays for the detection and quantification of FOXP3 (Hs00174114_m1) and the housekeeping gene glyceraldehyde 3-phosphate dehydrogenase (GAPDH) were purchased from Applied Biosystems and used according to the manufacturer's protocol. The Applied Biosystems 7500 fast real-time PCR system was used, all samples were run in duplicates and each run contained several negative controls (no template) and a reference sample. There were no significant differences in cycle threshold neither within nor between the experiments. Quantification of gene expression was performed according to the comparative cycle threshold method (2^−ΔΔCT^) after validating the experiments by comparing the amplification efficiencies of targets and housekeeping gene. All samples were normalized to GAPDH and compared to a reference sample used in all the experiments

### Transmigration Assay

Transmigration was assessed in a well established assay [20]–[22]. Using 3-µm pore-size membranes (BioCoat™ fibronectin coated cell culture inserts (BD Biosciences)). Membranes were rehydrated with RPMI 1640 for one hour at 37°C. 10^6^ PBMCs or CD4+ T cells suspended in 1 ml of RPMI 1640 plus 2.5% fetal calf serum (FCS, PAA, Pasching, Austria), were added to the upper chamber. The lower compartment was filled with 1,5 ml of RPMI 1640 supplemented with 10% FCS. After 12 hours at 37°C, contents of the lower chamber were collected and processed for flow cytometry. The frequency of CD4+ and CD4+Foxp3+ T cells was determined before migration (baseline) and within the migrated fraction.

### Treg suppression assay

Suppressive function of Tregs was assessed by using an optimized autologous suppression assay [23], [24]. CD4+CD25 negative cells (responder) were stained with carboxyfluorescein succinimidylester (CFSE) using the Vybrant®CFDA-SE Cell Tracer Kit (Invitrogen, Karlsruhe, Germany). Anti-CD3/anti-CD28 coated beads (Dynabeads, Invitrogen, Karlsruhe, Germany were used as a stimulus. Stimulus-responder and responder-Treg ratios were carefully titrated to achieve suboptimal suppression. 5×10^4^ CFSE labelled CD4+CD25− were cocultured with 5×10^3^ CD4+CD25^high^CD127^low^ or the same number of irradiated CD4+CD25− as a control for unspecific suppression and Dynabeads (Invitrogen, Oslo, Norway) at a ratio of 1 bead per 50 cells in the absence or presence of Natalizumab.

To investigate suppression in the presence of antigen-presenting cells, autologous CD4+CD25− and CD4+CD25^high^CD127^low^ T cells were cocoultured with allogeneic immature and mature dendritic cells at a T cell to DC ratio of 5 to 1 in the presence of 2,5 µg/ml anti-CD3 (clone: UCHT1, Ebioscience, San Diego, USA) and 2,5 µg/ml anti-CD28 (clone: CD28.2, Ebioscience, San Diego, USA) in 96 well plates precoated with 5 µg/ml aCD3 for at least 2 hours at 37°C. After 96 hours the percentage of proliferating responder cells as reflected by CFSE dilution was assessed by flow cytometry. The percentage of proliferating cells in the presence of Tregs was normalized to proliferation of responder cells in the presence of an equal number of irradiated non-regulatory T cells.

### Statistics

All data sets were analyzed using student's t-test. *, p<0.05; **, p<0.005

## Supporting Information

Figure S1 The relative decrease in CD49d immunoreactivity on CD4+ T cells under Natalizumab therapy is remarkably diminished after development of persistent neutralizing antibodies in 1 patient with RRMS. CD49d surface expression was analyzed by flow cytometry before (M0), one month after the first infusion (M1) and 6 months after initiation of therapy (M6). Neutralizing antibodies were first detected 16 weeks after the first infusion and confirmed in the re-test 6 weeks later.
(0.43 MB TIF)

Figure S2 (A) Intracellular Foxp3 levels after human T regulatory cell purification using MACS technology (left panel) or fluorescence activated cell sorting using CD4 and CD25 as markers (right panel). Histograms show the purity of the populations yielded by this isolation procedures (light grey: CD4+CD25− effector cells, dark grey: CD4+CD25high, one representative example is shown). (B) Example of fluorescence activated flow cytometric cell sorting of Tregs using CD4, CD127 and CD25 as markers (left). This strategy yields a highly pure population of Foxp3 expressing T regulatory cells as evidenced by intracellular staining for the transcription factor Foxp3 (histogram, light grey CD4+CD25− effector cells, dark grey: CD4+CD127lowCD25high).
(1.42 MB TIF)
