# Correction: Effects of Natalizumab Treatment on Foxp3+ T Regulatory Cells

**DOI:** 10.1371/annotation/d0ea7ce8-836e-428d-bdfa-5d07bc1b3af2

**Published:** 2010-12-28

**Authors:** Max-Philipp Stenner, Anne Waschbisch, Dorothea Buck, Sebastian Doerck, Hermann Einsele, Klaus V. Toyka, Heinz Wiendl

The authors would like to disclose the following Competing Interests: Dr. Wiendl has received funding for travel and speaker honoraria from Bayer Schering Pharma, Biogen Idec/Elan Corporation, Sanofi-Aventis, Merck Serono, and Teva Pharmaceutical Industries Ltd.; has served/serves as a consultant for Merck Serono, Medac, Inc., Sanofi-Aventis/Teva Pharmaceutical Industries Ltd., Biogen Idec, Bayer Schering Pharma, Novartis, and Novo Nordisk; and receives research support from Bayer Schering Pharma, Biogen Idec/Elan Corporation, Sanofi-Aventis, Merck Serono, and Novo Nordisk. The study was partly supported by funds from BiogenIdec, the manufacturer of the monoclonal antibody Natalizumab (Tysabri).

