# Supplementary figures and images for: Effects of Natalizumab Treatment on Foxp3+ T Regulatory Cells
Source: PLoS One. 2008 Oct 6;3(10):e3319. doi: 10.1371/journal.pone.0003319 (PMC2553177; doi:10.1371/journal.pone.0003319)

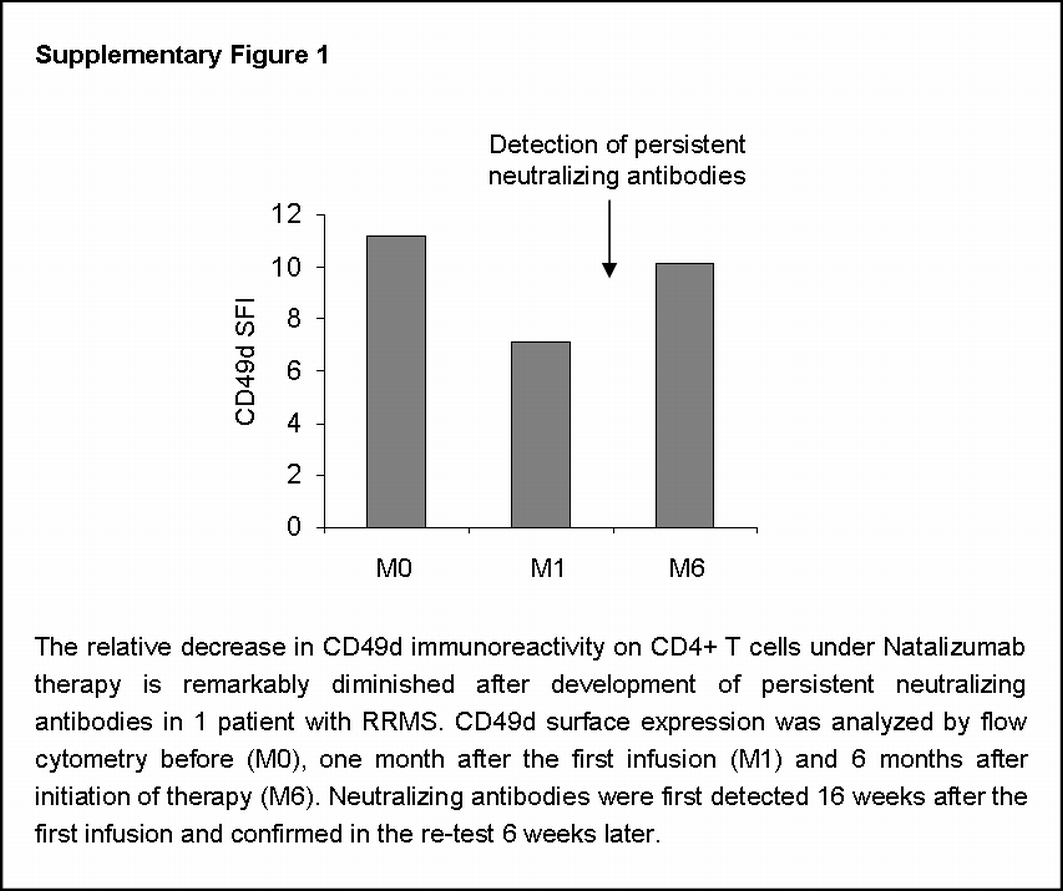

Supplement: Figure S1 — The relative decrease in CD49d immunoreactivity on CD4+ T cells under Natalizumab therapy is remarkably diminished after development of persistent neutralizing antibodies in 1 patient with RRMS. CD49d surface expression was analyzed by flow cytometry before (M0), one month after the first infusion (M1) and 6 months after initiation of therapy (M6). Neutralizing antibodies were first detected 16 weeks after the first infusion and confirmed in the re-test 6 weeks later. (0.43 MB TIF) [file pone.0003319.s001.tif]

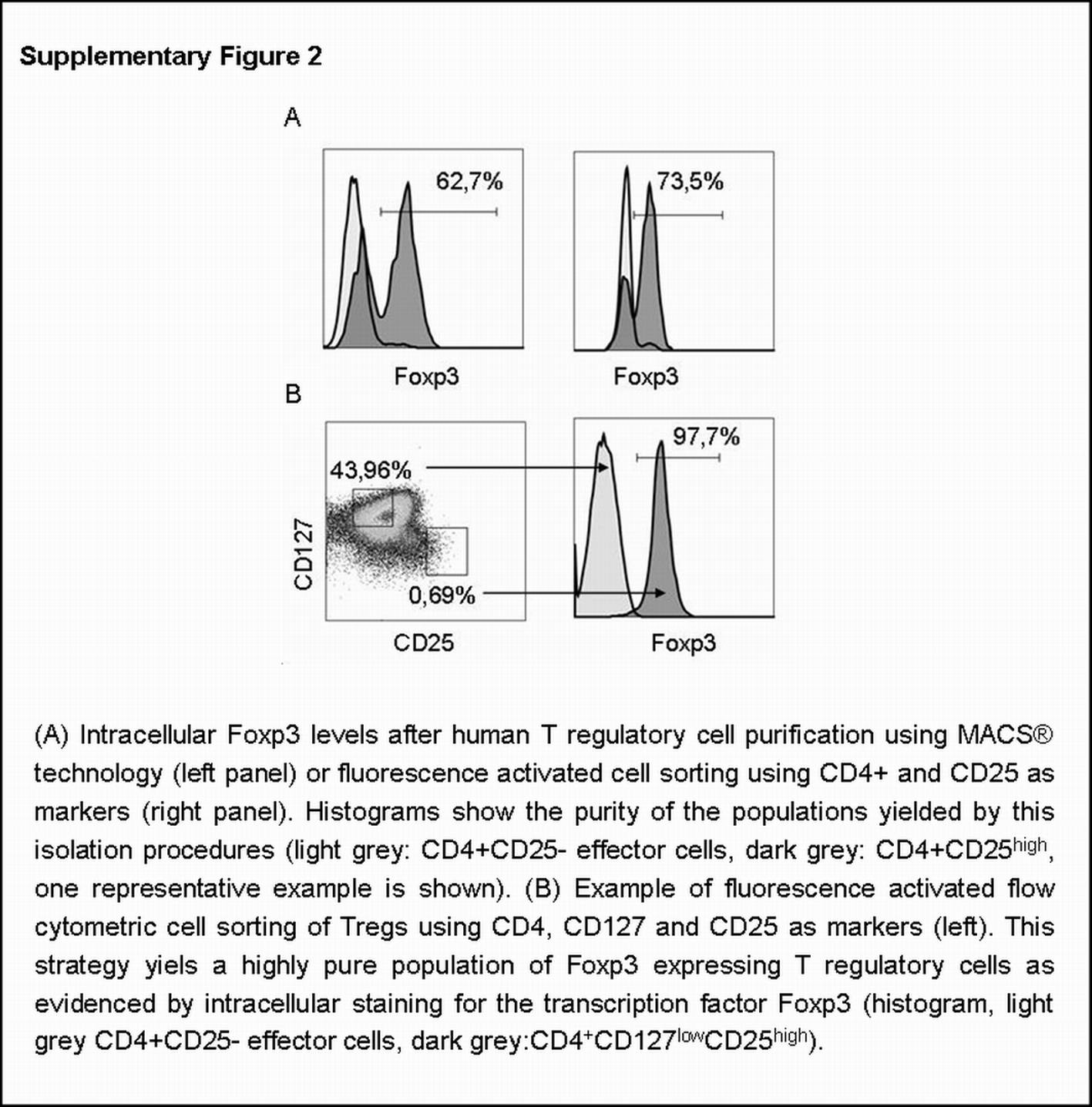

Supplement: Figure S2 — (A) Intracellular Foxp3 levels after human T regulatory cell purification using MACS technology (left panel) or fluorescence activated cell sorting using CD4 and CD25 as markers (right panel). Histograms show the purity of the populations yielded by this isolation procedures (light grey: CD4+CD25− effector cells, dark grey: CD4+CD25high, one representative example is shown). (B) Example of fluorescence activated flow cytometric cell sorting of Tregs using CD4, CD127 and CD25 as markers (left). This strategy yields a highly pure population of Foxp3 expressing T regulatory cells as evidenced by intracellular staining for the transcription factor Foxp3 (histogram, light grey CD4+CD25− effector cells, dark grey: CD4+CD127lowCD25high). (1.42 MB TIF) [file pone.0003319.s002.tif]
